# Characterization of a Digestive α-Amylase in the Midgut of *Pieris brassicae* L. (Lepidoptera: Pieridae)

**DOI:** 10.3389/fphys.2016.00096

**Published:** 2016-03-15

**Authors:** Ali Sharifloo, Arash Zibaee, Jalal J. Sendi, Khalil Talebi Jahroumi

**Affiliations:** ^1^Department of Plant Protection, Faculty of Agricultural Sciences, University of GuilanRasht, Iran; ^2^Department of Plant Protection, College of Agriculture and Natural Resources, University of TehranKaraj, Iran

**Keywords:** *Pieris brassicae*, α-amylase, purification, acarbose, gene expression

## Abstract

The current study deals with a digestive α-amylase in the larvae of *Pieris brassicae* L. through purification, enzymatic characterization, gene expression, and *in vivo* effect of a specific inhibitor, Acarbose. Although α-amylase activity was the highest in the whole gut homogenate of larvae but compartmentalization of amylolytic activity showed an equal activity in posterior midgut (PM) and anterior midgut (AM). A three step purification using ammonium sulfate, Sepharyl G-100 and DEAE-Cellulose Fast flow revealed an enzyme with a specific activity of 5.18 U/mg, recovery of 13.20, purification fold of 19.25 and molecular weight of 88 kDa. The purified α-amylase had the highest activity at optimal pH and temperature of 8 and 35°C. Also, the enzyme had *V*_*max*_ values of 4.64 and 3.02 U/mg protein and *K*_*m*_ values of 1.37 and 1.74% using starch and glycogen as substrates, respectively. Different concentrations of acarbose, ethylenediamine tetraacetic acid, and ethylene glycol-bis (β-aminoethylether) N, N, N′, N′-tetraacetic acid significantly decreased activity of the purified α-amylase. The 4th instar larvae of *P. brassicae* were fed on the treated leaves of *Raphanus sativus* L. with 0.22 mM of Acarbose to find *in vivo* effects on nutritional indices, α-amylase activity, and gene expression. The significant differences were only found in conversion efficiency of digested food, relative growth rate, and metabolic cost of control and fed larvae on Acarbose. Also, amylolytic activity significantly decreased in the treated larvae by both biochemical and native-PAGE experiments. Results of RT-PCR revealed a gene with 621 bp length responsible for α-amylase expression that had 75% identity with *Papilio xuthus* and *P. polytes*. Finally, qRT-PCR revealed higher expression of α-amylase in control larvae compared to acarbose-fed ones.

## Introduction

Carbohydrates gained from plant tissues must be broken down being absorbable via epithelial cells of insect midgut. There are several digestive carbohydrases in insects which are categorized into two groups based on their specific substrates, (i) des-polymerases which hydrolyse internal bonds of polysaccharides such as starch and glycogen and (ii) Glycosidases involved in catalysis of oligo- and di-saccharide hydrolysis (Terra and Ferreira, 2012). α-Amylase (α-1,4-glucan 4-glucanohydrolase, EC 3.2.1.1) is one of the des-polymerases catalyzing α-1,4-glucan bonds in starch and glycogen (Terra and Ferreira, 2012). Structure of α-amylase contains three conserved domains; Catalytic core domain (= Domain A) is the active site with three acidic terminal as two aspartic acid and one glutamic acid (Machinus et al., 1995) while domains B and C are oppositely on each side of domain A (Strobl et al., 1998). Insect α-amylases are dependent on calcium and chloride ions for structural integrity and activity (Kaur et al., 2014). In physiological status, α-amylases improve digestive performance of insects leading to survival within different living conditions and increase their biological fitness (Kaur et al., 2014).

The large cabbage butterfly, *Pieris brassicae* L. (Lepidoptera: Pieridae) is one of the key pests of many agricultural crops in Europe, North Africa, and Asia because of its extensive migration ability and feeding habit (Johnson and Triplehorn, 2004). Adults are the white butterflies with specific black spots that lay their eggs on several species of Brassicaceae family like cabbage, radish, rapeseed, and etc. The hatched larvae intensively feed on leaves of host plants leading to complete defoliation and feces smudges. The damaged crops fail to yield and become additionally infested with bacteria and fungi (Zibaee, 2012). Chemical spraying with synthetic insecticides is the most common control against *P. brassicae*. Chemical spraying seems to be an efficient control but the effects on environment, human, and non-target organisms are of significant concerns. So, providing a host-plant resistant program is of interest to achieve an efficient and safe control. In case, targeting of α-amylase might be a suitable way to decrease population outbreaks of *P. brassicae* with the lowest side-effects. Six different classes of α-amylases have been reported as lectin-like, knottin-like, cereal-type, Kunitz-like, c-purothionin-like, and thaumatin-like (Franco et al., 2002) highlighting potential roles to get transgenic plants. But it must be noted that providing such a transgenic plants requires precise and sufficient knowledge on the biochemical properties of insect α-amylases and interactions with host plants or specific inhibitors (Kaur et al., 2014). In the current study, several experiments were carried out determining α-amylase importance in digestive process of the 4th instar larvae of *P. brassicae* as (i) compartmentalization of α-amylase activity in different preparations of larval midgut, (ii) purification of the enzyme by a three step purification, (iii) characterization of the purified α-amylase such as optimal pH and temperature as well as *in vitro* responses to specific inhibitors, (iv) *in vivo* effects of acarbose on nutritional indices and amylolytic activity, and (v) determination of the gene responsible for α-amylase secretion and its expression in response to larval feeding on acarbose.

## Materials and methods

### Insect rearing

The eggs of *P.brassicae* were collected from radish fields in northern Iran, transferred to laboratory and placed in sterile containers (20 × 10 × 5 cm) provided with wet cotton at 25 ± 2°C of temperature, 85% of relative humidity and 16 Light:8 Dark of photoperiod. The hatched larvae were fed on radish leaves in the same sized containers. The rearing conditions were daily checked, containers were cleaned and new leaves provided until molting to the 4th instar (Zibaee, 2012).

### Dissection and sample preparations

The 4th instar larvae of *P. brassicae* were randomly selected and dissected in ice cold saline solution (NaCl, 125 mM). Samples including total, anterior- and posterior-midguts were separately homogenized by a glass pestle in ice cold distilled water and centrifuged at 28,500 g for 20 min (Elpidina et al., 2001). Supernatant was considered as the soluble fraction and the amount of protein was determined based on Lowry et al. (1951). Pellets from the first centrifugation were used to solubilize the membrane-bound enzyme in Triton X-100 in a ratio of 10 mg per mg of protein. The pellets were incubated with Triton X-100 for 20 h at 4°C. Then, those were centrifuged at 28,500 g for 30 min and gained supernatant was used as membrane-bound fraction for α-amylase assay (Ferreira and Terra, 1983). Moreover, secretion fraction refers to the sample obtained after cutting of midgut. On the other hand, when we longitudinally cut the midgut, a solution exited out that we considered it as secretion fraction. Provided samples were poured in dialysis membrane and put in saline solution (125 mM) to remove saline from the samples (Elpidina et al., 2001; Mehrabadi et al., 2009).

### Compartmentalization of α-amylase activity

Based on Bernfeld (1955), 20 μL of the prepared samples was separately added to a mixture including 80 μL of universal buffer (Containing Glycine, succinate, and 2-morpholinoethan sulfuric acid, 20 mM, pH 9, Frugoni, 1957; Zibaee, 2012) and 40 μL of Starch (1%) prior to incubation at 30°C for 30 min. Then, 100 μL of dinitrosalicylic acid (DNS) was added to the mixture and heated in boiling water for 15 min. After cooling, the absorbance was read at 545 nm. The blank samples contained buffer, substrate, DNS, and distilled water instead of enzyme. Also, the negative control was considered for all sample preparations using pre-boiled enzyme for 30 min. For further experiment, a concentration of acarbose (3 mM, prepared in distilled water) was used besides all reactions mixture in a separate experiment to presence and activity of α-amylase in the different preparations of the larval midgut. In details, 50 μL of starch (1%) was added to 100 μL of universal buffer, then 30 μL of acarbose (3 mM) was added and incubation was done for 10 min. The enzymatic incubation was initiated after adding 20 μL of enzyme solution (separately from all mentioned midgut-preparations) and prolonged for 30 min. To stop the reaction, 100 μL of DNS was added and the tubes containing reaction mixtures (including blank) were put in boiling water for 10 min. Finally, 100 μL of each reaction mixture was poured into microplate wells and absorbance was read at 545 nm.

### α-amylase purification

Purification of α-amylase was done based on Englard and Seifter (1990) and Dennison (1999). The total larval midgut of *P. brassicae* was prepared as mentioned earlier and the crude extract (30 mL) was precipitated with ammonium sulfate (30 and 70%) at 4°C. The precipitates were collected by centrifugation at 6000 g for 15 min, then were diluted in 5 mL of universal buffer and dialyzed overnight at 4°C against the same buffer. The dialyzed sample was applied to a Sepharyl G-100 column, equilibrated with universal buffer (20 mM, pH 9, Zibaee, 2012). The column was run at a flow rate of 0.5 mL/min. In each collected sample, the amount of protein and the amylolytic activity were measured and the fractions showing highest enzymatic activity were pooled and applied to a diethylaminoethyl (DEAE)-cellulose column, equilibrated with universal buffer (20 mM, pH 9, Zibaee, 2012). The enzyme was eluted at a flow rate of 0.5 mL/min with a linear NaCl gradient (0–0.6 M). Fractions (1.5 mL/tube) were collected and the protein concentration and the α-amylase activity were determined as previously described. Fractions showing highest amylolytic activity were pooled and used in the enzymatic characterization.

### Molecular weight and purity of the α-amylase

Sodium dodecylsulphate polyacrylamide gel electrophoresis (SDS-PAGE) was run according to Laemmli (1970). The acrylamide concentrations were 10% for the separating gel and 4% for the stacking gel. After running the gel at 100 mV as a constant voltage, proteins on the polyacrylamide gel were stained with 0.2% Coomassie brilliant blue R-250. Then, the stained protein was compared with a molecular mass standard containing beta-galactosidase (120 kDa), bovine serum albumin (85 kDa), ovalbumin (50 kDa), cardonic anhydrase (35 kDa), beta-lactoglobulin (25 kDa), lysozyme (20 kDa) (Pierce™ Prestained Protein MW Marker; #26612). A second gel as native-PAGE was also run to find amylolytic activity in the purified sample (Without SDS). When dye containing samples reached at bottom of glass, gel was immersed in Triton X-100 (1%) for 30 min and incubated in a solution containing universal buffer (20 mM, pH 9, Zibaee, 2012), 10 mM of NaCl and 2 mM of CaCl_2_ and starch (1%) for 3 h. On the other hands, a 100 ml solution was prepared containing all the mentioned components. Finally, the gel was washed and stained with a solution containing 1.5% of Iodide and 3% of KOH. The white band in dark background indicates amylolytic activity.

### Determination of optimal pH and temperature in the purified α-amylase

The effect of different pH sets on the purified α-amylase of *P. brassicae* larvae were determined using universal buffer (20 mM) from 4 to 12 ranges (Frugoni, 1957). The purified enzyme were added to a mixture containing starch (1%) and universal buffer and incubated for 30 min. Afterward, 100 μL of DNS was added to stop the reaction and the tubes containing reaction mixtures were put in boiling water. Finally, absorbance was read at 545 nm. The effect of temperature on amylolytic activity was determined by incubating the reaction mixture (Determined optimal pH) at the following temperatures for 30 min: 20, 25, 30, 35, 40, 45, 50, 55, and 60°C.

### Kinetic parameters of the purified α-amylase

Kinetic parameters of the purified α-amylase were determined using different concentrations (0.2-0.5-0.4-0.6-0.8-1%) of starch and glycogen as substrates. The enzymatic assay was done as described earlier; maximum velocity (*V*_*max*_) and constant of Michealis-Menten (*K*_*m*_) were estimated by Sigma plot software, version 6.

### Effect of specific inhibitors on the purified α-amylase activity

Different concentrations (0.1-0.5-0.7-1-3-5 mM) of specific inhibitors including acarbose, ethylenediamine tetraacetic acid (EDTA), ethylene glycol-bis (β-aminoethylether) N, N, N′, N′-tetraacetic acid (EGTA), triethylenetetramine hexaacetic acid (TTHA), and diethyldithiocarbamate (DETC) were prepared and separately added to a mixture containing 30 μL of the purified enzyme, 20 μL of Starch (1%), and 50 μL of universal buffer (20 mM). After 30 min, 100 μL of DNS was added and the absorbance was read at 545 nm. Also, IC_50_ concentrations of the inhibitors on the amylolytic activity were calculated by POLO-PC software.

### *In vivo* assay of acarbose

Five pieces of radish leaves (4 × 4 cm) were immersed in Triton X-100 (0.02%) solution as control and the IC_50_ concentration of acarbose (0.22 mM, calculated based on in vitro experiment and POLO-PC) for 10 s, then those were put on a filter paper (Whatman No. 1) to be dried for 30 min. The larvae were weighed and fed on the treated leaf (above) separately and the experiment continued for 72 h. Weight of the larvae, feces and leave remnants were weighed every day and fresh leaves were provided for each larvae. The nutritional indices were calculated as described by Scriber and Slansky (1981) as: Conversion efficiency of ingested food (ECI) estimates percentage of ingested food that is converted to biomass and it was calculated as: [biomass gained (mg fresh mass)/food ingested (mg dry mass)] × 100. Conversion efficiency of digested food (ECD) estimates the efficiency which digested food is converted to biomass, it and was calculated as: biomass gained (mg fresh mass)/[food ingested (mg dry mass)–feces (mg dry mass)] × 100. Approximate digestibility (AD) estimates the amount of ingested food that is digested, and it was calculated as: [food ingested (mg dry mass)–feces (mg dry mass)]/food ingested (mg dry mass) × 100. Consumption Index (CI) = E/A, Relative Consumption Rate (RCR) = E/(W_0_ × T) and Relative Growth Rate (RGR) = P/(W_0_× T) were also determined besides Metabolic cost (MC) as 100–ECD. Ten larvae in three replicates were used for each treatment and live larvae were dissected for α-amylase assay (See the above explained procedure) and gene expression (See below).

### Gene expression of α-amylase

#### RNA extraction

Guanidine/phenol solution was used to extract total RNA from the midgut of P. brassicae larvae followed by feeding on control and acarbose treated leaves. RNA extraction was performed by adding 1 ml of ice cold RNX-PLUS solution to the tubes containing homogenized samples. The tubes were vortexed for 5–10 s and incubated at room temperature for 5 min. The procedure was continued by adding 200 μL of chloroform and mixed well by shaking for 15 s, then the mixture was incubated on ice for 15 min prior to centrifugation at 20,000 g for 15 min at 4°C. Aqueous phase was transferred to new RNase-free 1.5 ml tubes and added equal volume of isopropanol, gently mixed and incubated on ice for 15 min. Then the mixture was centrifuged at 25,000 g for 15 min at 4°C. The supernatant was removed and 1 ml of 75% ethanol was added and tubes were centrifuged at 9500 g for 8 min at 4°C. The supernatant was discard and let the pellets to dry at room temperature for a few minutes. Finally, the pellets were dissolved in 50 μL of DEPC (diethylpyrocarbonate)-treated water.

#### RNA quantity and quality determination

The estimation of RNA integrity was performed by UV/VIS spectrophotometer at multiple wavelengths. The assessment of RNA was carried out by 50 μL of DEPC treated water and 1 μL of extracted RNA into cuvette, then the absorbance was read at optical density (OD) of 260 nm to find the ratio of OD 260/280 as the quality, OD 260/240 or OD 260/320 as the purity and the extraction performance. An OD 260/280 ratio greater than 1.8 is usually considered an acceptable indicator of RNA quality.

#### cDNA synthesis

cDNA synthesis was done by a Thermo Scientific RevertAid First Strand Cdna Synthesis Kit. Synthesis of first-strand cDNA was done in a total reaction volume of 20 μL containing 4 μL of 5X reaction buffer for reverse transcriptase, 1 μL (20 U/μL) RiboLock™ RNase Inhibitor, 2 μL (10 Mm) dNTP Mix, 1 μl (200 U/μL) RevertAid™ H Minus Reverse Transcriptase, 1 μL Oligo-dT primers, 11 μL DEPC-treated water, and 1 μL RNA. The tubes were incubated 60 min at 42°C. Finally, the reaction was terminated by heating at 70°C for 10 min. The reverse transcription reaction product can be directly used in second strand cDNA synthesis or stored at −20°C.

#### cDNA amplification

The cDNA was amplified by polymerase chain reaction (PCR) using a specific forward primer 5′- GGTTTCAGAATTGACGC-3′ and a specific reverse primer 5′- GCGATCTGGTTGCTG -3′ by the estimated product length of 630 bp. The primers were designed based on the blast of available α-amylase sequences of butterflies at NCBI (National Center for Biotechnological Information) using Clastal Omega software (http://www.ebi.ac.uk/Tools/msa/clustalo/). PCR was performed for 30 cycles at a denaturing temperature of 95°C for 2 min, 95°C for 30 s, at an annealing temperature of 51°C for 30 s, 72°C for 30 s and at an extending temperature of 72°C for 5 min in a tube with 25 μl containing 1 μl cDNA templates, 2.5 μl reaction buffer, 0.5 μl dNTPs, 0.75 μL MgCl_2_, 0.25 μL Taq polymerase, 19 μL DEPC treated water, and 0.5 μL of forward and reverse primer in a thermocycler.

#### Gene expression

Expression of α-amylase gene was analyzed using real-time, quantitative PCR in the acrarbose treated- and control larvae, separately. qPCR experiment was performed by a Maxima SYBR Green/ROX Kit (FERMENTAS Co). The amplifications were carried out using three technical replicates of 12.5 μL reaction volumes containing 6.25 μL the SYBR Green PCR Master Mix, 0.5 μL of forward (5′- TGAGTACACAGCGTTAGCCG-3′) and reverse primer (3′- CCTCTTTGGTTGTCGTGGTT -5′) [10 mM, Product length, 171 bp] designed from gained sequence of α-amylase (http://workbench.sdsc.edu/), 3.3 μL Nuclease-Free Water and 1 μL of cDNA. The thermal cycling conditions were performed at temperature of 95°C for 2 min, 95°C for 30 s, 51°C for 30 s, 72°C for 30 s, and finally of 72°C for 5 min. Control gene in qRT-PCR was 18srRNA which amplified using a forward primer 5′- CACGGGAAATCTCACCAGG-3′ and a reverse primer 3′- CAGACAAATCGCTCCACCAACTA-5′ suggested by Lu et al. (2013).

#### Relative gene expression analysis calculation using a ΔΔCt method

The relative quantification in gene expression was determined using the 2^−ΔΔCt^ method (Livak and Schmittgen, 2001). First, Ct values of technical replicates were averaged. Then, ΔCt was calculated by normalizing Ct (Target gene) to Ct (Reference gene). Next, average ΔCt values were calculated for the control treatment. ΔΔCt was calculated by normalizing ΔCt from Cq (Target gene) to Cq (Reference gene) to average ΔCt for the treatment control. Relative gene expression was calculated (Step 5) as 2^−ΔΔCt^ (Livak and Schmittgen, 2001).

#### Sequence analysis and homology modeling

Sequence analyses were performed with the BLAST program of the National Centre for Biotechnology Information (NCBI), NIH, Bethesda, MD, USA. The phylogenetic analysis was performed using Gene-Dock and Mega softwares.

#### Protein determination

Protein concentration was determined by a kit provided by Ziest Chem (www.zeistchem.com) based on Lowry et al. (1951)'s method using bovine serum albumin as standard.

#### Statistical analysis

All data were compared by one-way analysis of variance (ANOVA) followed by Tukey's studentized range distribution when significant differences were found at *p* ≤ 0.05 except for data of nutritional indices which compared by t-test. Differences between samples were considered statistically significant at *p* < 0.05 and marked in figures and tables with letters and asterisks.

## Results

### Compartmentalization of α-amylase activity

**Figure 1:**
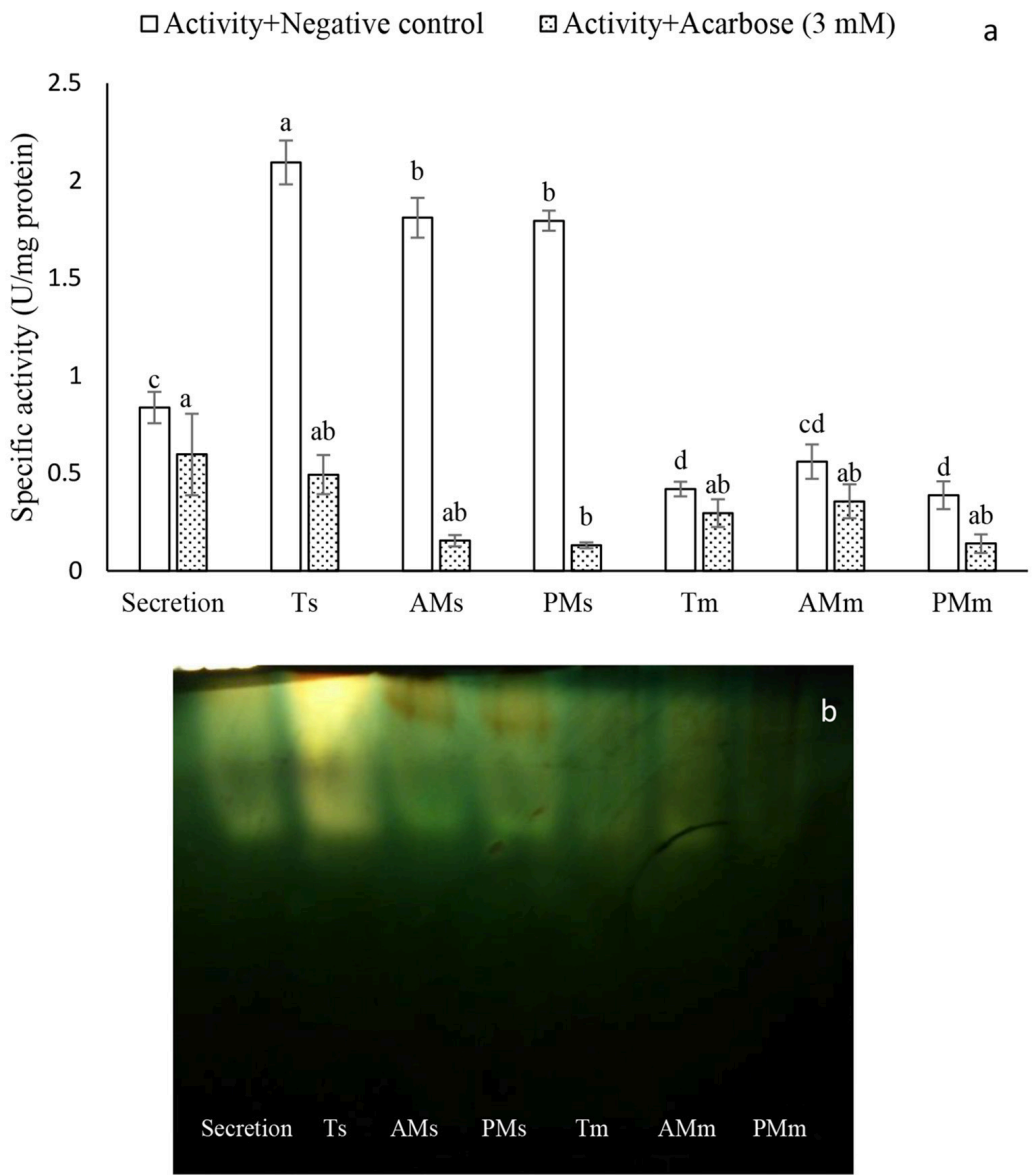
**Compartmentalization of α-amylase activity in the larvae of *Pieris brassicae*. (A)** Determination of amylolytic activity using negative control and Acarbose (3 mM). Abbreviations refer to: Ts, Total midgut solution; AMs, Anterior Midgut solution; PMs, Posterior Midgut solution; Tm, Total midgut membrane-bound; AMm, Anterior Midgut membrane-bound, Posterior Midgut membrane-bound. **(B)** Native-PAGE of the amylolytic activity. Statistical differences have been marked by different letters (Tukey test, *p* ≤ 0.05).

Although amyloytic activity was found in all midgut preparations of P. brassicae larvae, the soluble fraction of the total, posterior-, and anterior midgut preparations showed the highest amylolytic activity compared to other preparations (Figure 1A). Meanwhile, significant inhibition of α-amylase was observed in the preparations treated by 3 mM concentration of acarbose as specific inhibitor (Figure 1A). In native-Page, amylolytic bands were observed and their disappearance indicated inhibition of acarbose on α-amylase in the midgut preparations of P. brassicae larvae (Figure 1B).

### α-amylase purification

**Figure 2:**
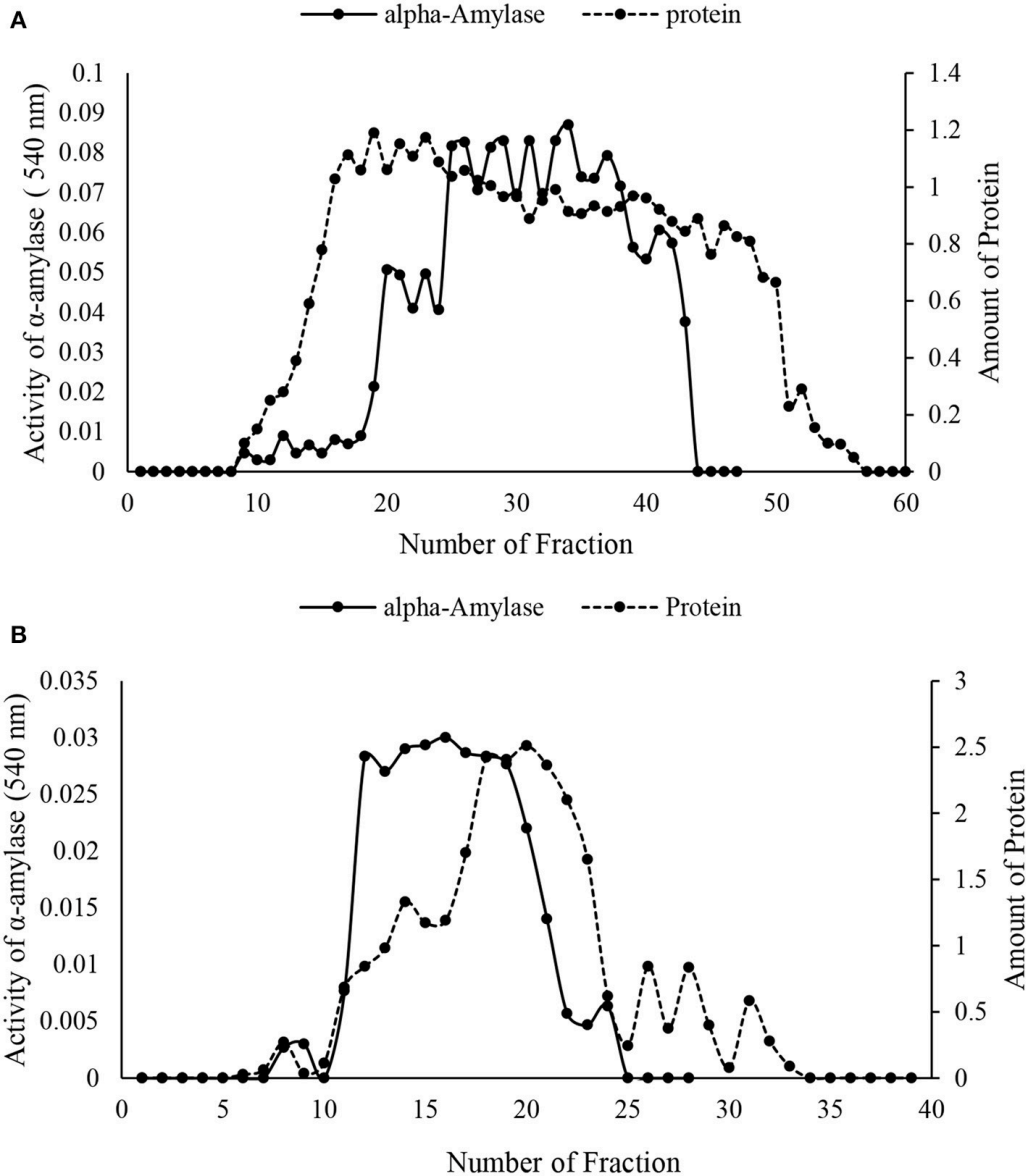
**Column chromatography of α-amylase in the midgut of Pieris brassicae larvae. (A)** Sepharyl G-100 chromatography of the samples from ammonium precipitation (70%). (B) DEAE-fast flow chromatography (ion exchange) by samples showing the highest amylolytic activity from Sepharyl G-100. All steps were carried out at 4°C.

**Figure 3:**
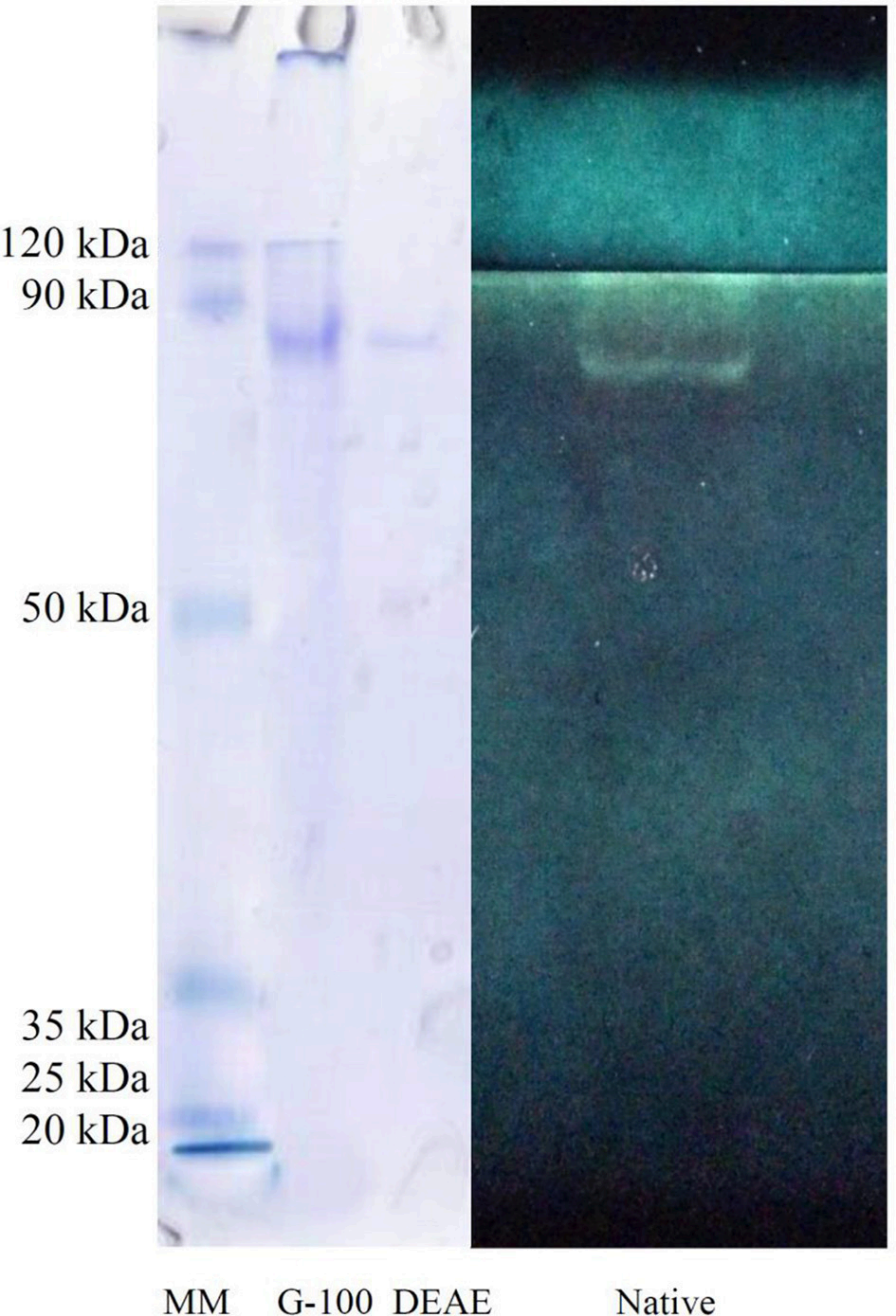
**Polyacrylamide gel electrophoresis (PAGE) of α-amylase in Pieris brassicae larvae showing purity, molecular weight (SDS-PAGE), and quality of the purified enzyme (Native-PAGE)**.

The crude preparation of the larval midgut was precipitated with the two concentrations of ammonium sulfate indicating specific activity of 1.28 U/mg protein, recovery of 43.39% and purification fold of 4.75 at the end of the step (Table 1). Afterward, dialyzed sample was loaded into sepharyl G-100 column for further purification. Sixty fractions were taken in which fractions 26–39 showed the highest α-amylase activity (Figure 2A). These fractions were pooled by considering specific activity of 4 U/mg protein, recovery of 16.98% and purification fold of 14.86 (Table 1). In ion exchange chromatography using DEAE-Cellulose Fast flow, 40 fractions were collected and fractions 12–19 showed the highest amylolytic activity as specific activity of 5.18 U/mg protein, recovery of 13.20%, and purification fold of 19.25 (Figure 2B, Table 1). These fractions were pooled to determine purity, molecular weight, and amylolytic activity by PAGE procedures. In SDS-PAGE, a single band was found with molecular weight of 88 kDa (Figure 3). Native-PAGE revealed amylolytic activity of the purified enzyme with a single white band in dark background (Figure 3).

**Table 1:** **Purification of α-amylase in the larval midgut of ***Pieris brassicae*****.

**Purification steps**	**Unit activity (U) ± SE**	**Amount of protein (mg)**	**Specific activity (U/mg protein) ± SE**	**Recovery (%)**	**Purification fold**
Crude extract	1.06±0.071	3.93	0.269±0.018	100	1
Ammonium sulfate (30%)	0.50±0.056	1.80	0.277±0.031	47.16	1.03
Ammonium sulfate (70%)	0.46±0.059	0.592	1.28±0.101	43.39	4.75
Sepharyl G-100	0.18±0.01	0.045	4±0.234	16.98	14.86
DEAE-Fast flow	0.14±0.012	0.027	5.18±0.458	13.20	19.25

^*^All steps were carried out at 4°C.

### Determination of optimal pH and temperature in the purified α-amylase

**Figure 4:**
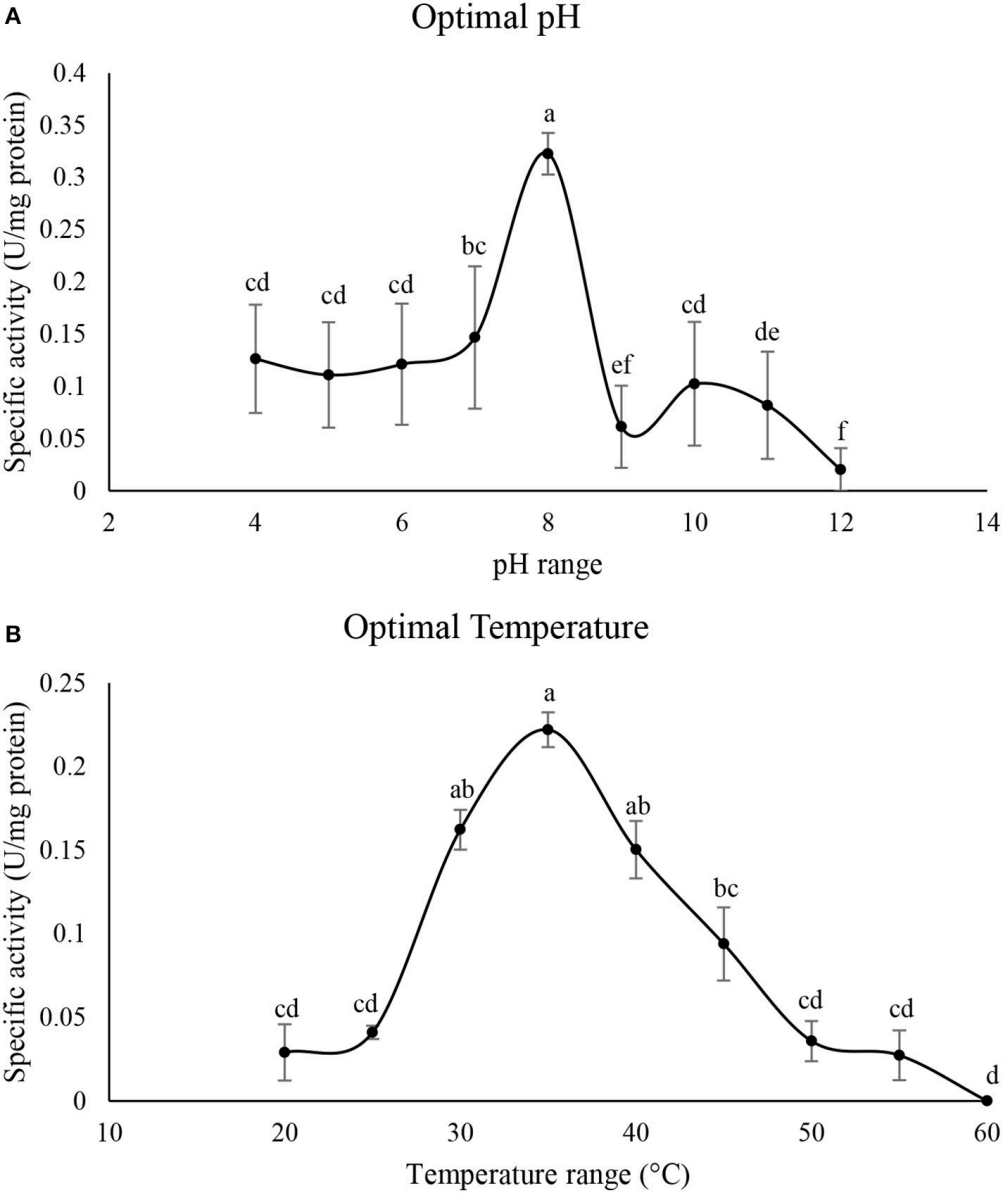
**Determination of optimal pH (A) and temperature (°C) (B) in the purified α-amylase of Pieris brassicae larvae**. Statistical differences have been marked by different letters (Tukey test, *p* ≤ 0.05).

Figure 4A shows optimal pH of the purified α-amylase in P. brassicae larvae. The enzymatic activity was steady from pH values of 4–7 then it sharply increased at pH 8 showing the highest α-amylase activity prior to lowering (Figure 4A). Also, activity of the purified α-amylase increased from 25to 35°C then it decreased to 60°C with the optimal temperature of 35°C (Figure 4B).

### Kinetic parameters of the purified α-amylase

**Figure 5:**
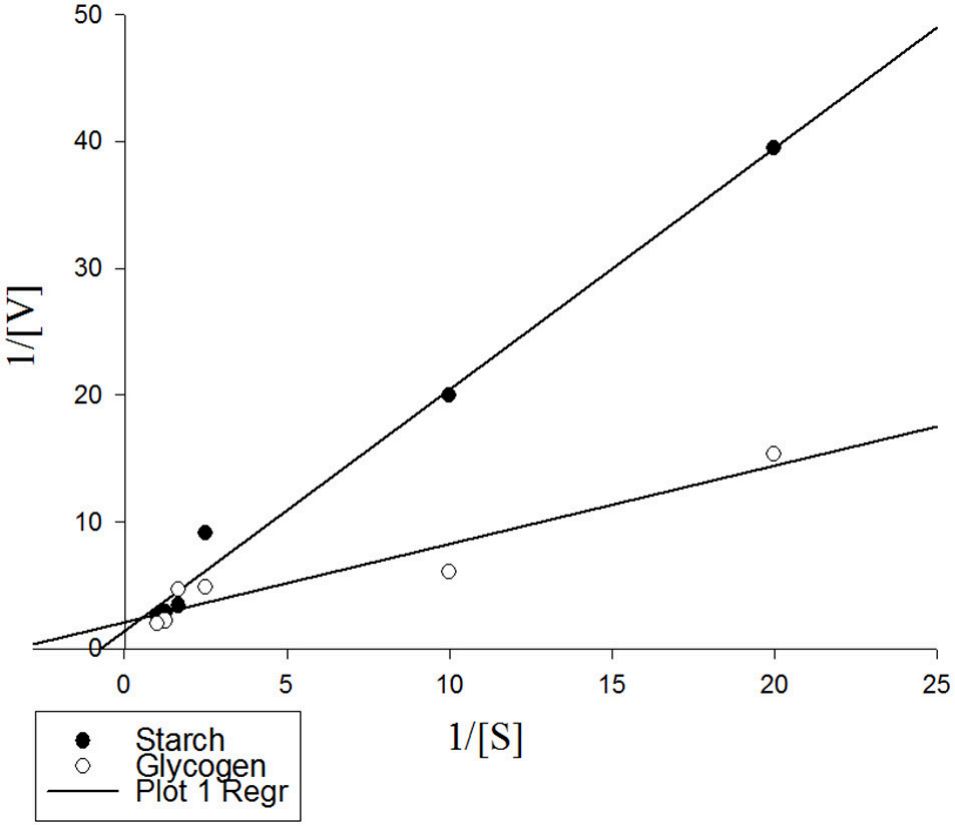
**Lineweaver-Burk analysis showing kinetic parameters of the purified α-amylase from Pieris brassicae larvae on starch and glycogen as substrates**. Data were inserted in Sigma Plot software version 6 and kinetic parameters were determined as V_max_: 7.24 U/mg protein, K_m_: 1.37 for starch, and V_max_: 0.47 U/mg protein, K_m_: 1.74 for glycogen. R2 for both treatments were 0.989 and 0.913, respectively.

Linweaver-Burk analysis was used to calculate kinetic parameters of the purified α-amylase using starch and glycogen as substrates (Figure 5). The enzyme had V_*max*_ values of 4.64 and 3.02 U/mg protein and K_*m*_ values of 1.37 and 1.74% using starch and glycogen as substrates, respectively (Figure 5).

### Effect of specific inhibitors on the purified α-amylase activity

**Figure 6:**
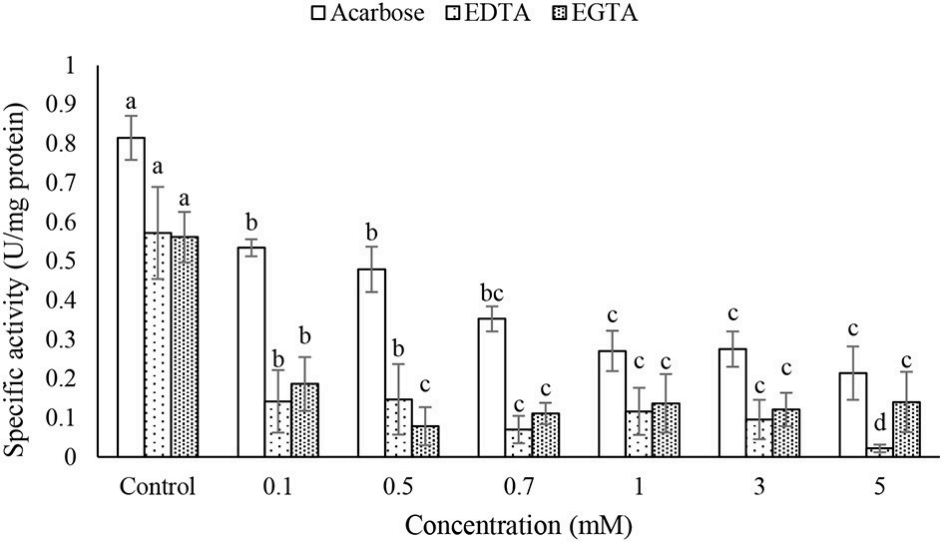
**Effects of acarbose and chelating agents (EDTA and EGTA) on activity of the purified α-amylase in the midgut of Pieris brassicae larvae**. Statistical differences have been marked by different letters (Tukey test, *p* ≤ 0.05).

Different concentrations of acarbose, EDTA, and EGTA significantly decreased activity of the purified α-amylase in a dose-dependant manner but TTHA and DETC had no effects on the amylolytic activity (Figure 6). Moreover, IC_50_ concentration of acarbose found to be 0.22 mM.

### *In vivo* assay of acarbose

**Figure 7:**
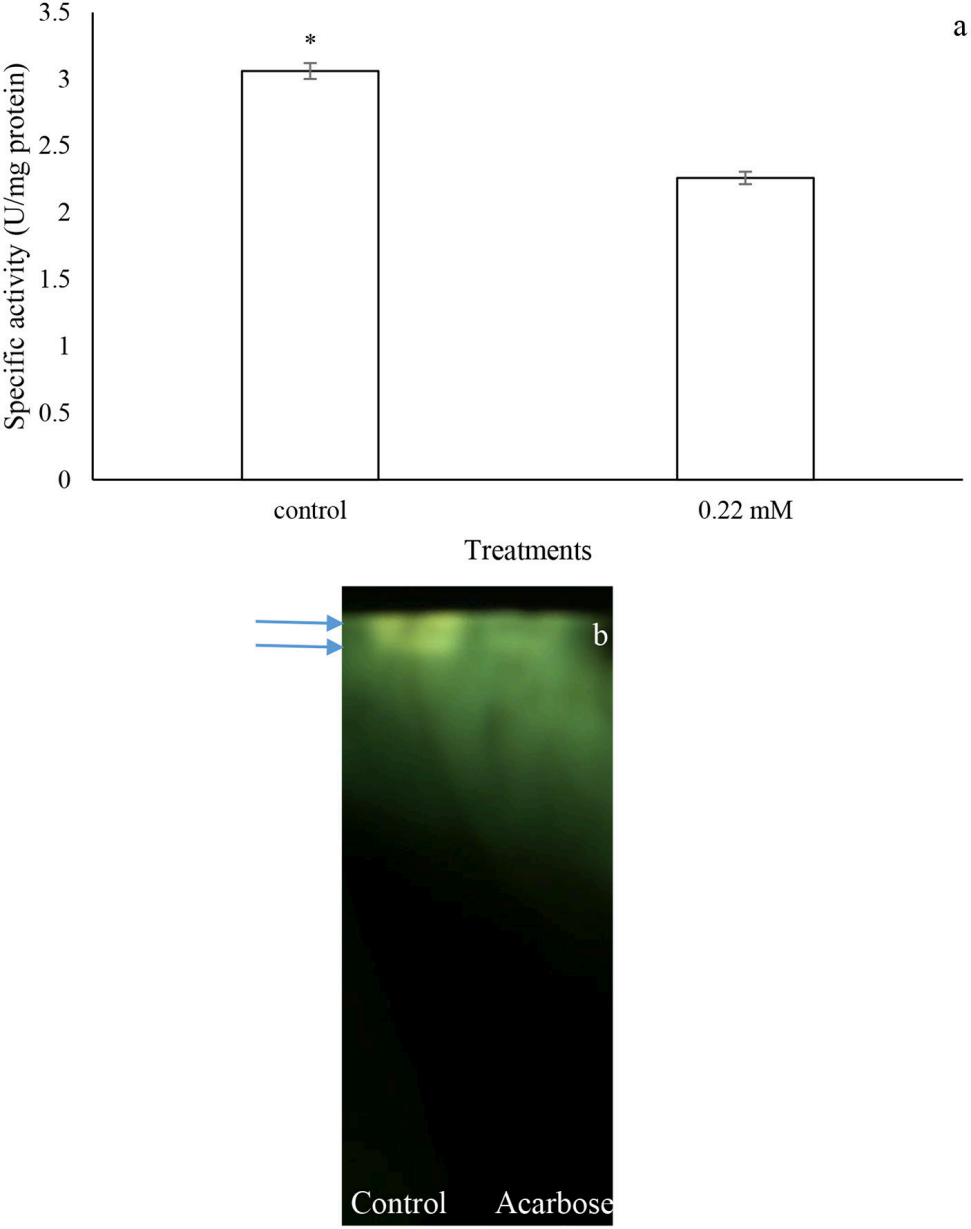
**In vivo effects of acarbose on α-amylase activity of Pieris brassicae larvae. (A)** Biochemical assay. (B) Native-PAGE. Statistical differences have been marked by asterisk (t-test, *p* ≤ 0.05). Arrows refer to bands.

Larvae of P. brassicae were fed on the leaves treated with acarbose (0.22 mM) and distilled water as control to examine changes in nutritional indices and α-amylase activity. The statistical differences were found in the amounts of ECD, RGR, and MC between control and acarbose fed larvae (Table 2). Also, activity of α-amylase in the fed larvae on acarbose was significantly lower than that of control larvae (Figure 7). Also, sharpness of the amylolytic band in native-PAGE decreased compared to control (Figure 7B).

**Table 2:** **Nutrition indices of Pieris brassicae larvae fed on the IC_50_ concentration of Acarbose**.

**Nutritional indices**	**AD**	**ECI**	**ECD**	**CI**	**RCR**	**RGR**	**MC**
Control	67.73	15.51	24.15^*^	15.87	1.55	1.56^*^	61.75
0.22 mM	65.57	13.69	21.65	16.03	1.68	0.51	78.34^*^

*Statistical differences have been shown by asterisks (t-test, p ≤ 0.05)*.

*AD, approximate digestibility; ECI, efficiency of conversion of ingested food; ECD, efficiency of conversion of digested food; CI, consumption index; RCR, relative consumption rate, RGR, relative growth rate; MC, Metabolic Cost*.

### Gene expression of α-amylase

**Figure 8:**
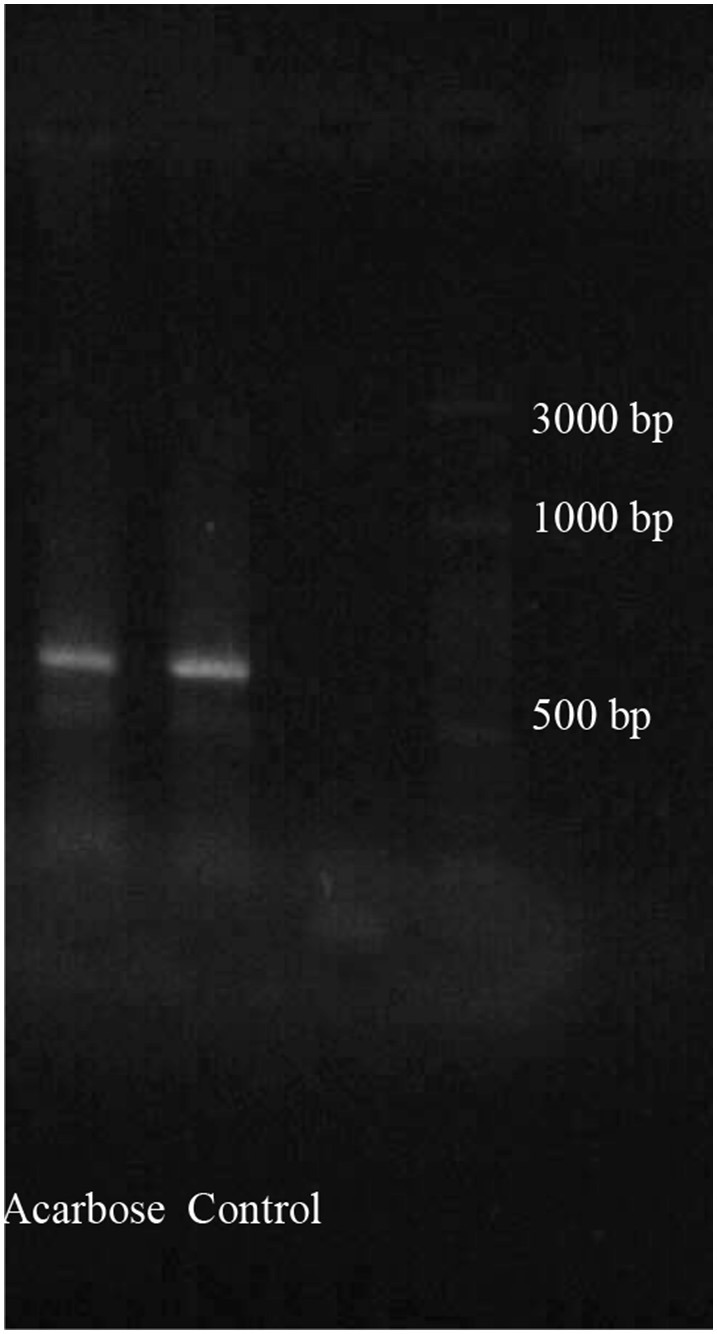
**PCR products from total larval mRNAs of Pieris brassicae fed on control and acarbose treated leaves**. Determination of cDNA goodness using specific primers for α-amylase gene amplification. PCR product show the relative length between 600 and 700 bp of ladder.

**Figure 9:**
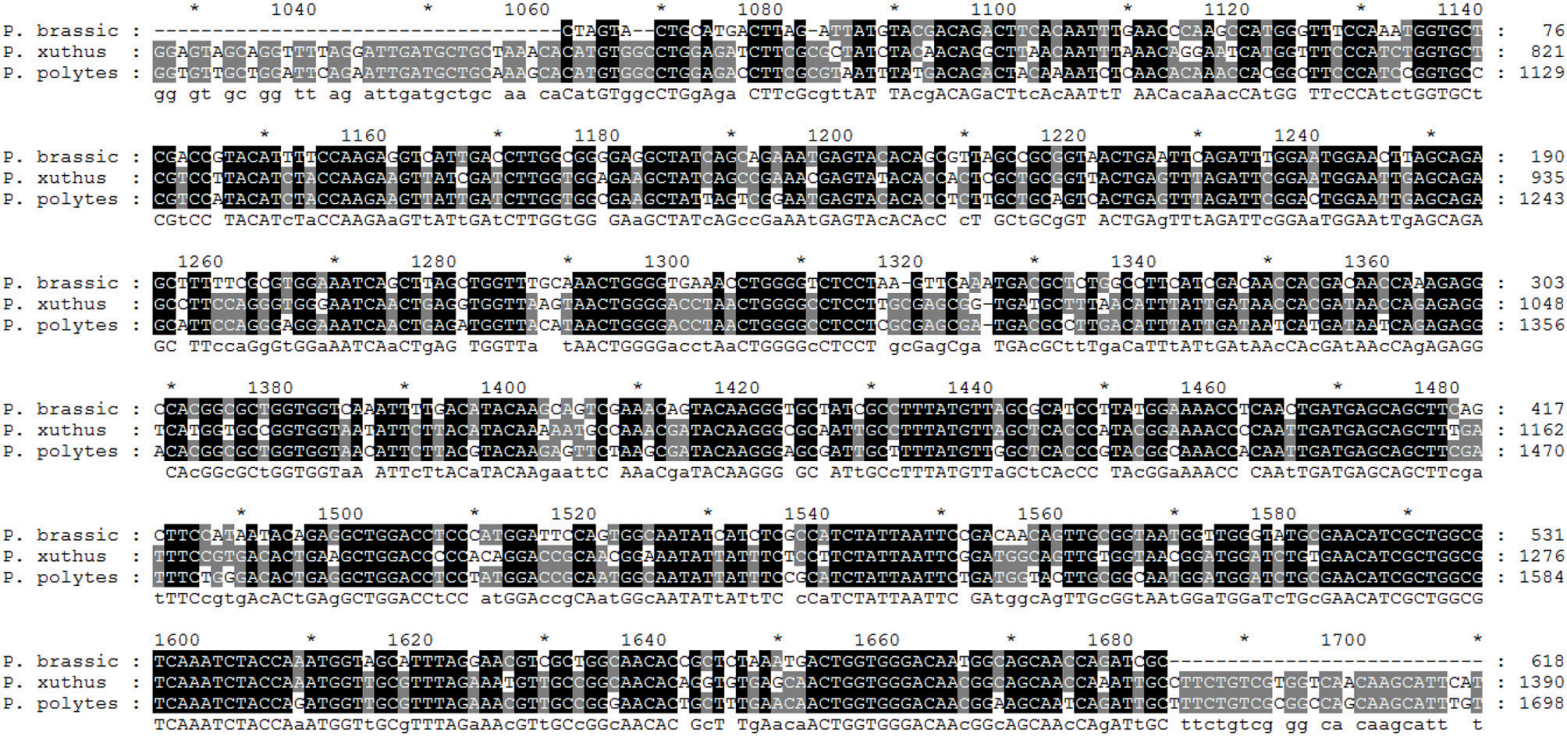
**Sequencing result and alignment of PCR products proved to be α-amylase of P. brassicae larvae by GENEDOC software**. The given sequence was aligned with the α-amylases of Papilio xuthus and Papilio polytes as the most similar sequences based on NCBI database. ^*^Refers to the same base in sequences.

**Figure 10:**
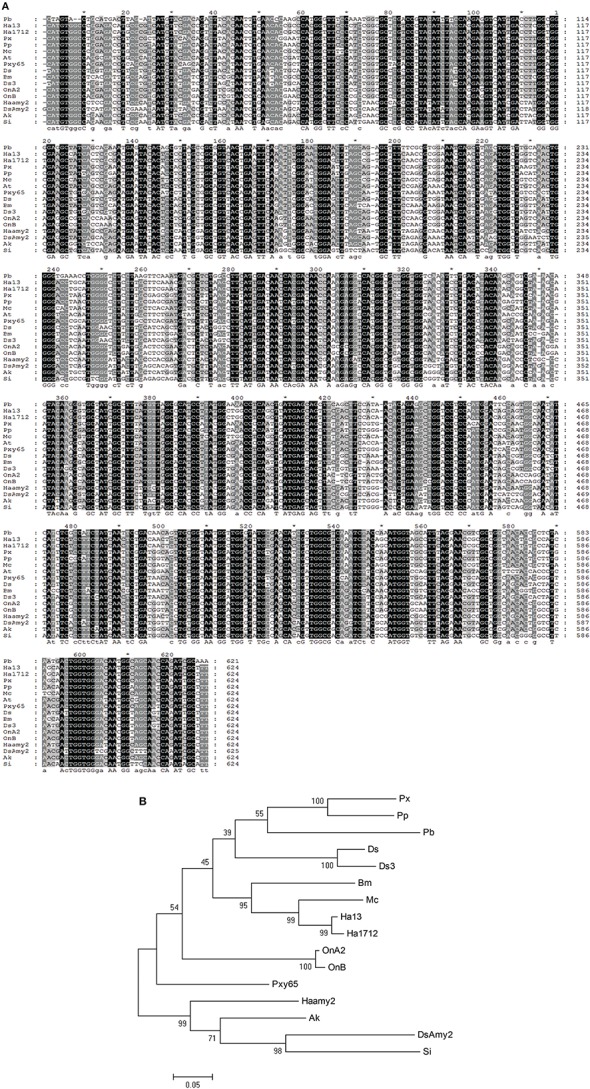
**Phylogenetic tree of the α-amylase from P. brassicae with other lepidopterans. (A)** Alignment results in Genedoc software. **(B)** Phylogenetic tree drawn by Mega software. ^*^Refers to the same base in sequences. Px, Papilio xuthus; Pp, Papilio polytes; Pb, Pieris brassicae; Ds, Diatraea saccharalis alpha-amylase 1; Ds3, Diatraea saccharalis alpha-amylase 3; Bm, Bombyx mori; MC, Mamestra configurata; Ha13, Helicoverpa armigera GH13Amy-1; Ha1712, Helicoverpa armigera HaFLS01712; OnA2, Ostrinia nubilalis alpha-amylase A2; OnB, Ostrinia nubilalis alpha-amylase OnB; Pxy65, Plutella xylostella LOC105380365; Haamy2, Helicoverpa armigera alpha-amylase2; AK, Amyelois transitella; DSAmy2, Diatraea saccharalis alpha-amylase2; Si, Scirpophaga incertulas.

Initially, total RNAs were extracted from control and fed larvae on acarbose and cDNAs were immediately synthesized. Then, the genetic region responsible for α-amylase expression was amplified by the specific primers mentioned in Materials and Methods section (Figure 8). Sequencing of the obtained 620 bp product revealed similarity of the amplified region with the reported α-amylase of Papilio xuthus and Papilio polytes (Figure 9). A phylogenetic tree was drawn using mega software in the maximum likelihood option indicating identity of 71–75% (Figures 9, 10). Finally, relative expression of the α-amylase gene in the control and fed larvae on acarbose found to be 2.06-fold which showed statistical lower expression of the enzyme in acarbose treated larvae (t-test, *p* ≤ 0.05).

## Discussion

Both insect larvae and adults requires carbohydrates for energetic demands, growth, longevity, movement, and reproduction (Nation, 2008; Chapman, 2012). Herbivorous insects utilize plant tissues full of carbohydrates (Mainly starch and glycosides) which are digested by the activities of different carbohydrases to provide monomers like glucose to be absorbed via epithelial cells (Nation, 2008). α-Amylases are one of the important classes of digestive enzymes that break down starch within plant tissues to oligosaccharides prior to be further hydrolyzed to glucose by glucosidases (Kaur et al., 2014). Also, other physiological roles than digestion may be considered for α-amylases because they are active during non-feeding stages like pupal stage (Zhu et al., 2005). α-Amylases have been characterized in different orders of insects e.g., Orthoptera, Hemiptera, Heteroptera, Hymenoptera, Diptera, Lepidoptera, and Coleoptera orders and the findings have cleared different aspects of their physiological roles in insects (Kaur et al., 2014). In our study, assay of α-amylase in different midgut preparations of P. brassicae larvae revealed the higher enzymatic activity in the soluble fraction rather than membrane-bound fraction although anterior- and posterior-midgut preparations showed similar activities of α-amylase. Studies on localization of amylolytic activity in Panesthia cribrata Saussure (Blattodea: Blaberidae), Nauphoeta cinerea Oliver (Blattoptera:Blaberidae), and Lutzomyia longipalpis (Diptera: Psychodidae) indicated the higher activity of α-amylase in soluble and anterior midgut preparations (Scrivener et al., 1989; Elpidina et al., 2001; Vale et al., 2012). Also, in the hemipterans like Euygaster integriceps Puton (Hemiptera: Scutelleridae), Brachynema germari Kolenati (Hemiptera: Pentatomidae), and Andrallus spinidens Fabricius (Hemiptera: Pentatomidae) which have a four sectioned midgut, the higher amylolytic activity reported in the third soluble fraction of the midgut (Mehrabadi et al., 2009; Ramzi and Hosseininaveh, 2010; Sorkhabi-Abdolmaleki et al., 2014). Fialho et al. (2012) demonstrated the highest activity of α-amylase in the anterior-midgut of Podisus nigrispinus (Dallas) (Hemiptera: Pentatomidae). The author concluded that digestion of food in P. nigrispinus may be initiated by α-amylase and continued by other carbohydrases. There are several hypotheses regarding distribution of digestive α-amylases in different regions of the midgut. First, a regulated release of the enzyme to lumen has been reported by stored vesicles in the cytoplasm so that a slight enzymatic activity can be found during starvation (Lehane et al., 1996). Second, different activities of α-amylases in anterior- and posterior-midgut may be referred to their protection from proteolysis by endogenous enzymes which enable insects to efficiently utilize food resources with a low metabolic expenditure (Elpidina et al., 2001). Third, pH gradient in the midgut is one of the main physico-chemical mechanisms providing amylolytic compartmentalization to increase digestive efficiency and interferences with other carbohydrases (Elpidina et al., 2001; Vale et al., 2012).

A three-step purification led to isolating a molecule with the specific amylolytic activity of 5.18 U/mg protein, recovery of 13.20%, and purification fold of 19.25. Electrophoresis by both SDS- and native-PAGE revealed the molecular weight of 88 kDa with the amylolytic activity. The molecular weight of many insect α-amylases have been reported from 28 to 87 kDa (Ferreira and Terra, 1983), but the values in Orius insidiosus and L. longipalpis were found to be 132 and 103 kDa (Zeng and Cohen, 2000; Vale et al., 2012). In lepidopterans, molecular weight of α-amylase has been reported as 87 kDa for Spodoptera frugiperda Smith (Lepidoptera: Noctuidae) (Ferreira et al., 1994), 48 kDa for Erinnyis ello L. (Lepidoptera: Sphingidae) (Santos and Terra, 1986), 56 kDa for E. kuehniella (Pytelkova et al., 2009), 51.2, 55 kDa for Helicoverpa armigera Hubner (Lepidoptera: Noctuidae) (Bhide et al., 2015). It is believed that the enzyme with high molecular masses may bypass the peritrophic membrane of the larvae (Vale et al., 2012). Moreover, the molecular mass of an enzyme may depend on isoforms, activation state and species studied highlighting its evolutionary or compatibility properties regarding the physiological role and interaction with the exposed dietary molecules.

The pH of a biochemical reaction is one of the key factors affecting enzymatic efficiency because it leads to proper conformation of an enzyme by composing the ionizable groups in an appropriate form. Also, the critical role of pH can be achievable by the type of amino acid residues involved in catalysis of the enzymatic active site (Kaur et al., 2014). Temperature is another critical factor on enzymatic activity in a media. In fact, elevating the media temperature to optimal value enhances the rate of enzyme-catalyzing reactions due to the higher kinetic energy and collision frequency of the engaged molecules (Delkash-Roudsari et al., 2014). Although optimal pHs of insect α-amylases have been reported from 4 to 10, caterpillars have shown the highest activity in alkaline range e.g., Cameraria ohridella (Stygar et al., 2010), Chilo suppressalis (Zibaee et al., 2008), Glyphodes pyloalis (Yezdani et al., 2010), Ephestia kuehniella (Pytelkova et al., 2009), Tecia solanivora (Valencia-Jimenez et al., 2008), H. armigera (Ozgur et al., 2009). Dow (1986) and Chapman (2012) found that high pH of caterpillar's midgut is an adaptation to feed on the plant tissues rich in tannins to reduce possibility of their binding to the enzyme. Moreover, Terra and Ferreira (2012) believed that the higher activity of α-amylases in alkaline pH is due to selection pressure during evolution by presence of alkaline RNQ (Arg, Asn, and Gln)-type α-amylases in the digestive tract of lepidopterans. In our case, larvae of P. brassicae feed on different plant species so they must have alkaline pH in their midgut (Zibaee, 2012) to increase digestive efficiency by avoiding interactions with potential metabolic compounds in their host plants. In case of temperature, reported optimal values for activity of insect α-amylases are 30–60°C (Kaur et al., 2014) which our finding on P. brassicae was within the reported range.

Linweaver-Burk analysis were used to find kinetic parameters of the purified α-amylase in the larvae of P. brassicae in presence of the two substrates, starch and glycogen. The analysis revealed the statistical higher V_max_ and the lower K_m_ for starch vs. glycogen. Since K_m_ determines the affinity between enzyme and substrate, the lower values indicated more affinity between enzyme and substrate. Also, V_max_ refers to the number of substrate molecules converted into product regarding enzymatic saturation by substrate and time unit (Kaur et al., 2014). In contrast of our findings, Sharifi et al. (2011) and Zibaee et al. (2012) reported higher affinity of α-amylases in Xanthogaleruca luteola Muller (Coleoptera: Chrysomelidae) and A. spinidens toward starch and glycogen. Although Kaur et al. (2014) believe it may be due to the highly branched structure of glycogen, but we hypothesize this is due to evolutionary process to cause somehow an adaptability between α-amylases and meal of the insects. Since carnivorous insects utilize glycogen as the storage carbohydrate in their prey but herbivores take starch from plant tissues. So, their relevant α-amylase must be efficient on the main carbohydrates of their meals.

Several studies have shown that insect α-amylases are the metalloenzymes requiring metal ions for activity, structural stability, and integrity (Terra and Ferreira, 2012; Kaur et al., 2014). Also, chloride ion activates amylolytic process by displacement of optimal pH although other anions may do so depending on their ionic size (Terra and Ferreira, 2012). In our study, EDTA and EGTA significantly decreased activity of the purified α-amylase in P. brassicae larvae. Other specific chelating agents caused no statistical effects on the enzymatic activity. These results suggest an importance of Ca^2+^ to increase activity of P. brassicae α-amylase which is consistence with other studies (Zibaee et al., 2008; Ozgur et al., 2009; Yezdani et al., 2010; Delkash-Roudsari et al., 2014; Sorkhabi-Abdolmaleki et al., 2014). Besides chelating agents, different concentrations of acarbose were used to find their potential inhibition on the purified enzyme. Acarbose is a non-proteinaceous inhibitor of carbohydrases mainly α-amylase and α-glucosidase that even is used as anti-diabetic drug to treat type 2 diabetes mellitus (Asano, 2003). In vitro experiment revealed inhibition of the purified α-amylase of P. brassicae by different concentrations of acarbose in a dose-dependent manner so that the half maximal concentration (IC_50_) of acarbose on the enzyme was found to be 0.22 mM. Moreover, in vivo experiments by the larvae fed on the leaves treated by acarbose (0.22 mM) demonstrated significant inhibition of the larval α-amylase in both biochemical assay and gel electrophoresis. The effect of acarbose on the digestion of P. brassicae larvae was further demonstrated by alleviating the amounts of ECD, RGR and increasing metabolic cost. Finally, qRT-PCR results showed the higher amylolytic activity in the control larvae compared to acarbose-treated ones. Although inhibition of insect α-amylases has been reported by plant origin compounds, the recent advances in genetic engineering technology caused possibility to incorporate the non-proteinaceous inhibitors, e.g., acarbose, in a foreseeable future (Kinney, 2006). This provisional perspective can be achievable since acarbose have shown no significant effects on the biological control agents studied till now (Hubert et al., 2007; Kaufnerova et al., 2007).

Based on an obtained cDNA sequence of P. brassicae α-amylase, a phylogenetic tree was constructed in order to show its relationship with other lepidopteran α-amylases. Data (As NCBI blast software) demonstrated a highly phylogenetic relationship of P. brassicae α-amylase (Pb) with H. armigera (GH13) (sharing 76% nucleotide identity), H. armigera (1712) (sharing 75% nucleotide identity), P. xuthus (sharing 75% nucleotide identity), P. polytes (sharing 75% nucleotide identity), M. configurata (sharing 75% nucleotide identity), Amyelois transitella (sharing 75% nucleotide identity), Diatraea saccharalis (sharing 74% nucleotide identity), Bombyx mori (sharing 74% nucleotide identity), Ostrinia nubilalis (A2) (sharing 72% nucleotide identity), and O. nubilalis (B) (sharing 72% nucleotide identity). Other data given by NCBI blast revealed identity of at least 50% between α-amylases of lepidopteran larvae indicating their highly phylogenetic relationship.

In the current study, activity of an α-amylase was determined in the different midgut preparations of P. brassicae larvae then it was purified and characterized using biochemical and genetic approaches. The purified α-amylase had a high molecular weight and the expected pH optima in alkali. The enzyme was calcium dependent and inhibited by the specific non-proteinaceous inhibitor, acarbose. Also, reverse genetic partially revealed a gene with 621 bp length and high similarity with α-amylases of other lepidopterans mainly two species of Papilionidae. α-Amylases are the promising enzymes to be targeted by inhibitors from natural or synthetic origin. Since, these enzymes play crucial roles in carbohydrate utilization of insects, genetic engineering techniques are aimed to provide resistant varieties bearing α-amylase inhibitor genes. These varieties can be considered as an economic mean to reduce crop losses and to alleviate reliance on insecticides. So, precise characterization of the digestive enzymes like α-amylases must be done before achieving to the goal.

## Author contributions

All authors listed, have made substantial, direct and intellectual contribution to the work, and approved it for publication.

### Conflict of interest statement

The authors declare that the research was conducted in the absence of any commercial or financial relationships that could be construed as a potential conflict of interest.
